# C-C bond cleavage in biosynthesis of 4-alkyl-L-proline precursors of lincomycin and anthramycin cannot precede *C*-methylation

**DOI:** 10.1038/s41467-018-05455-3

**Published:** 2018-08-09

**Authors:** Zdenek Kamenik, Radek Gazak, Stanislav Kadlcik, Lucie Steiningerova, Vit Rynd, Jiri Janata

**Affiliations:** Institute of Microbiology, Czech Academy of Sciences, Videnska 1083, 142 20 Praha 4, Czech Republic

## Introduction

**Fig. 1:**
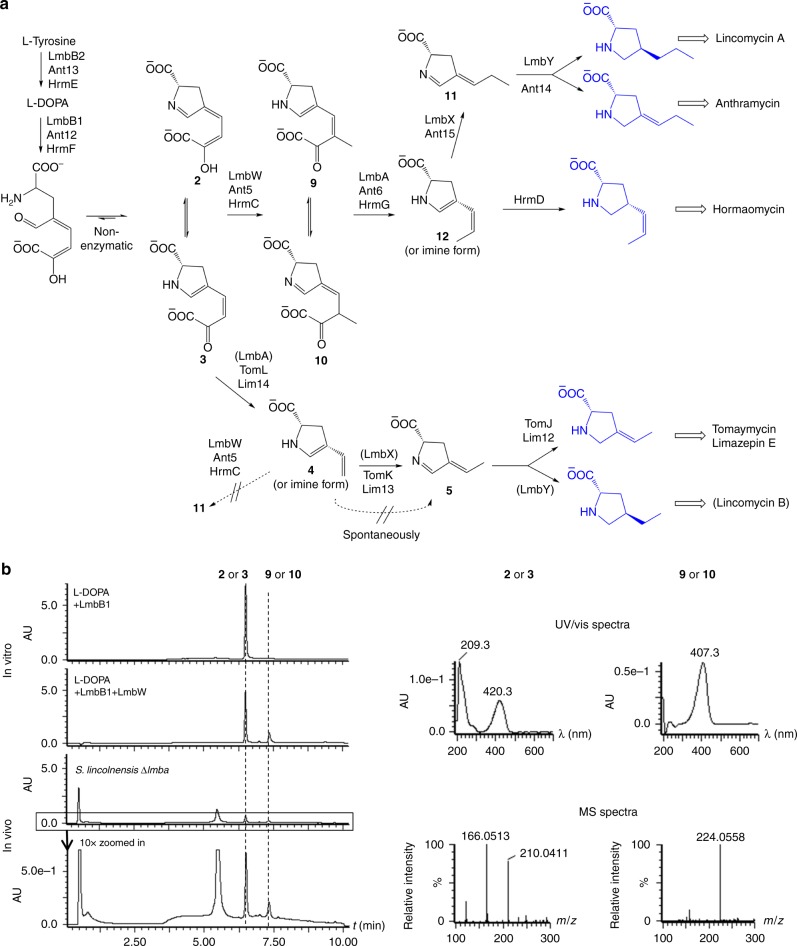
Biosynthetic steps catalyzed by LmbA/Ant6 and LmbW/Ant5 in the context of ALDP pathway. **a** Scheme of ALDP biosynthetic pathway (adopted from Jiraskova et al.^2^ and modified according to Kamenik et al.^10^); Dotted arrows indicate steps proposed by Zhong et al.^1^, brackets indicate a side-pathway, final ALDP precursors highlighted in blue are incorporated into the secondary metabolites. **b** In vitro (experiments from Jiraskova et al.^2^ re-examined using a more suitable chromatographic method) and in vivo (new experiments) *C*-methylation of **2**/**3** by LmbW; Chromatographic conditions: UPLC BEH Amide 1.7 µm, 2.1 × 50 mm column (Waters, USA), mobile phase: A-acetonitrile and B-50 mM ammonium acetate pH8:acetonitrile 1:1 (*v/v*), elution: 99% A for 2.5 min followed by a linear decrease from 99 to 1% A in 10 min, UV/VIS chromatograms extracted at 405 nm, MS spectra were recorded using an electrospray ionization technique in a negative mode

Zhong et al.^1^ confirmed that γ-glutamyltranspeptidase (γ-GTs) homologs are capable of cleaving a C–C bond, which was previously inferred by Jiraskova et al.^2^ in 2016 in a study based on gene inactivation experiments. The intriguing C–C bond cleavage catalyzed by LmbA and Ant6 γ-GT homologs from the biosynthesis of lincomycin A and anthramycin, respectively, was conclusively documented by Zhong et al.^1^. However, assignment of **2**/**3** as the LmbA and Ant6 substrate and **4**/**5** as the reaction product is questionable for several reasons; most importantly, it contradicts the current state of knowledge of the biosynthesis of 4-alkyl-l-proline derivatives (ALDP or APD used in previous literature; Fig. 1a)^2^. Here, we argue that LmbA/Ant6 γ-GT homologs do not utilize **2**/**3**, but intermediate **9**/**10**, which was previously proposed to be the main native substrate of LmbA^2^ and which is biosynthesized from **2**/**3** by a *C*-methylation reaction. Consequently, the main LmbA/Ant6 product is not **4**/**5** but compound **12**, which is a subject of isomerization in order to proceed towards the final ALDP of lincomycin A and anthramycin.

Here, we bring evidence that **2**/**3** is not the main native substrate of LmbA/Ant6 γ-GT homologs, but of LmbW/Ant5 *C*-methyltransferases. Indeed, we observed in vitro *C*-methylation of **2**/**3** by LmbW affording **9**/**10** and we also detected intermediate **9**/**10** in the cultivation broth of the Δ*lmbA* mutant of lincomycin producing strain *Streptomyces lincolnensis* (Fig. 1b). Even though the conversion of **2**/**3** into **9**/**10** by LmbW was only partial, it clearly showed that **2**/**3** serves as an LmbW/Ant5 substrate. To support that conversion of **2**/**3** by LmbW is not a side reaction resulting from broader substrate specificity of LmbW and that its main native substrate is indeed **2**/**3** and not **4**/**5** as the work by Zhong et al.^1^ suggests, we carried out a bioinformatic analysis of LmbW/Ant5. We found out that LmbW/Ant5 and their homologs (SibZ^3^, HrmC^4^, and Por10^5^) from the biosyntheses of other ALDPs are similar to ALDP-unrelated *C*-methyltransferases MppJ with known structure^6^ and MrsA^7^ (26% identity to LmbW according to BLAST for both MppJ and MrsA along the whole sequence; sequence alignment of LmbW and MppJ is available in Supplementary Fig. 1). MppJ and MrsA methylate phenylpyruvic and 5-guanidino-2-oxopentanoic acids, respectively, i.e., substrates structurally analogous to **2**/**3** and not **4**/**5**.

**Fig. 2:**
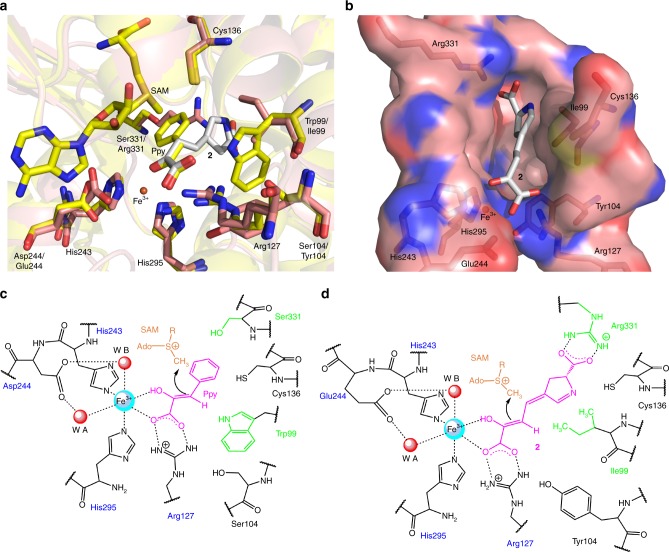
Comparison of the active sites and proposed reaction mechanism of MppJ and LmbW. **a** Comparison of active sites of MppJ (in yellow, crystal structure PDB ID: 4KIC [https://www.rcsb.org/structure/4KIC] with the substrates phenylenolpyruvate (Ppy) and *S*-adenosyl methionine (SAM)—adopted^6^) and LmbW (a homology model built using the MppJ structure and the SWISS-MODEL server^15^); LmbW is in pink; substrate **2** is in white. The positions of compound **2**, Fe^3+^, and SAM in the model were determined by superimposing the model on the 4KIC template in PyMOL^16^ and adjusting the position of **2** based on the position of the α-keto(enol)-carboxylic moiety of Ppy bound to MppJ. **b** Arrangement of the putative substrate binding pocket with **2** in the homology model of LmbW. **c** Schematic active site and a proposed mechanism of action of MppJ^6^, modified according to panel **a**. **d** Schematic active site and proposed mechanism of action of LmbW. Panels **c** and **d**: abbreviations of residues reflecting the common α-keto(enol)-carboxylic moiety of Ppy and **2** and the common proposed mechanism are in blue; abbreviations of residues differing in MppJ vs. LmbW, reflecting the uncommon moieties of Ppy vs. **2** (aromatic ring of Ppy vs. heterocyclic carboxylic moiety of **2**), are in green. Residue numbering corresponds to MppJ

Furthermore, methylation of phenylpyruvic acid catalyzed by MppJ is part of the biosynthesis of β-methyl-l-phenylalanine from l-phenylalanine^8^. Instead of direct methylation of l-phenylalanine, the machinery requires to proceed via phenylpyruvic acid, indicating the importance of the α-keto(enol)-carboxylic moiety of phenylpyruvic acid for the MppJ-catalyzed methylation. We propose that the same applies also to LmbW/Ant5 because their substrate **2**/**3** also contains the α-keto(enol)-carboxylic moiety. Importantly, conversion of the analogous substrates of MppJ and LmbW/Ant5 through a common reaction mechanism is supported by comparison of the active sites of MppJ (based on the protein crystal structure)^6^ vs. LmbW (based on a homology model) depicted in Fig. 2. The α-keto(enol)-carboxylic moiety appears to play an important role in fixation of the substrate within the active site not only in the case of MppJ, but also LmbW/Ant5. All these enzymes share the residues important for α-keto(enol)-carboxylic moiety fixation as well as the methylation (four residues depicted in blue in Fig. 2c, d). In contrast to **9**/**10**, intermediate **4**/**5** (proposed as the LmbA/Ant6 reaction product and thus the LmbW/Ant5 substrate by Zhong et al.^1^) does not possess the α-keto(enol)-carboxylic moiety for the substrate fixation in the active site.

Moreover, the methylation of **4**/**5** would have to proceed through a different mechanism than reactions catalyzed by MppJ and MrsA, which would be inconsistent with the high conservation of the key catalytic residues within the active sites of MppJ and LmbW/Ant5. Based on the above-mentioned arguments, we claim that **2**/**3** is first *C*-methylated by LmbW/Ant5 and the reaction product **9**/**10** is utilized as a substrate of LmbA/Ant6 γ-GT homologs. However, **2**/**3** can serve as a minor substrate of LmbA if the *C*-methylation step is omitted and lincomycin B^9^, a side product of lincomycin A biosynthesis, is formed. Similarly, **2**/**3** undergoes C–C bond cleavage if the *C-*methyltransferase is not encoded within the biosynthetic gene cluster, which applies to the biosynthesis of e.g., tomaymycin^10,11^ and limazepine E^12^ with a two-carbon side-chain ALDP (Fig. 1a). Therefore, Zhong et al.^1^ elucidated the unusual C–C bond cleavage function of LmbA/Ant6, but using other than the main native substrate.

Furthermore, Zhong et al.^1^ claim that **4**, which they propose to be the product of **2**/**3** cleavage by LmbA/Ant6, is prone to spontaneous isomerization into **5** (Fig. 1a). They observed this isomerization during their unsuccessful attempt to synthesize **4**. However, **4** was previously synthesized by Saha et al.^13^, it was structurally characterized by nuclear magnetic resonance (NMR) and used for enzymatic assays, but its spontaneous isomerization into **5** was not reported. Specifically, Saha et al.^13^ conducted a two-step deprotection of an analogous compound (methyl ester was used instead of *tert*-butyl ester) using LiOH for methyl ester hydrolysis and trifluoroacetic acid for Boc deprotection, affording **4**, not **5**. Therefore, we consider the formation of **5** during deprotection of **4’** observed by Zhong et al.^1^ to be caused by the used deprotecting method. Importantly, spontaneous isomerization of **4** into **5** would be also inconsistent with the function of putative isomerases LmbX/Ant15. They were assigned for enzymatic isomerization of **4** into **5** based on (1) the comparison of the hormaomycin structure and its biosynthetic gene cluster, which does not encode a homolog of LmbX^4^, and (2) the production profile of the Δ*lmbX* and Δ*lmbX*Δ*lmbW* mutants of lincomycin producing strain *S. lincolnensis*^2^. These data show that if the enzymatic isomerization step of **4** into **5** is not involved in the ALDP biosynthesis, **4** or its analog **12** with a three-carbon side-chain is after reduction of its endocyclic double bond incorporated into the final secondary metabolite.

In addition, analytical chemistry data for **5** obtained by Zhong et al.^1^ from enzymatic reaction of **2**/**3** with LmbA/Ant6 are not sufficient for unambiguous structural elucidation of this compound. Comparison of ^1^H NMR spectra of **5** obtained enzymatically and by chemical synthesis is complicated by partial overlap of the terminal methyl group signal by the signal of NH_4_OAc, which together with a relatively low quality of the spectrum complicates easy identification in the case of the enzymatic product. Without analogous comparison of at least ^13^C NMR spectra of **5** obtained from both sources, it is difficult to see their virtual identity. The expansion present in the spectrum of **5** from enzymatic reaction looks like an expansion from a different spectrum. Moreover, the signal at 2.00 ppm (expansion in spectrum a) should be a doublet, similarly as in the spectrum b. Another misleading point is also the chemical name of **5** in page 39 of Supplementary Information, in which its name corresponds to the structure of **4**.

In summary, considering also our arguments, work of Zhong et al.^1^ represents a crucial missing proof of the ALDP biosynthetic pathway puzzle, i.e., the role of γ-GT homologs in the cleavage of oxalate from **2**/**3** (for compounds with a two-carbon side-chain ALDP) or its methylated derivative **9**/**10** (for compounds with a three-carbon side-chain ALDP including lincomycin A and anthramycin). The subsequent step in anthramycin and lincomycin A biosynthesis presumably involves isomerization catalyzed by LmbX/Ant15 so that the pathway proceeds towards the final ALDP intermediate.^14^

## Data availability

Data supporting the findings of this work are available within the paper and its Supplementary Information file and from the corresponding author on request.

## Electronic supplementary material

Supplementary Information
