# Supplementary material for: C-C bond cleavage in biosynthesis of 4-alkyl-L-proline precursors of lincomycin and anthramycin cannot precede C-methylation
Source: Nat Commun. 2018 Aug 9;9:3167. doi: 10.1038/s41467-018-05455-3 (PMC6085390; doi:10.1038/s41467-018-05455-3)
Supplement: Supplementary file 1 — Supplementary Information [file 41467_2018_5455_MOESM1_ESM.pdf]

C-C bond cleavage in biosynthesis of 4-alkyl-L-proline precursors of lincomycin and anthramycin cannot precede *C*-methylation

Zdenek Kamenik, Radek Gazak, Stanislav Kadlcik, Lucie Steiningerova, Vit Rynd, Jiri Janata*
Institute of Microbiology, Czech Academy of Sciences, Videnska 1083, 142 20 Praha 4, Czech Republic;
*janata@biomed.cas.cz

SUPPLEMENTARY INFORMATION

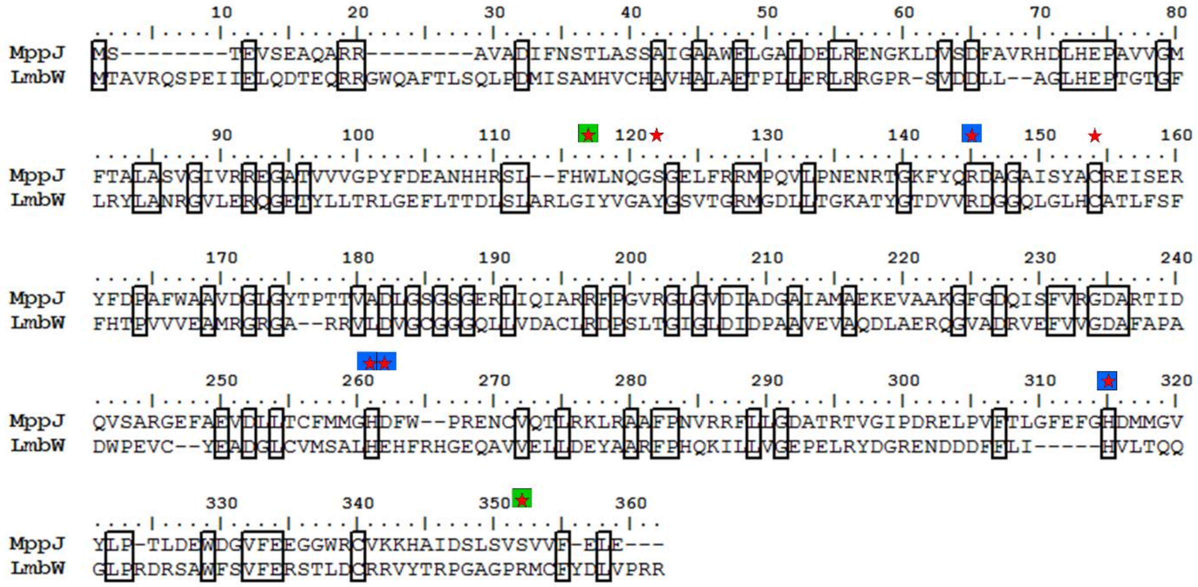

Supplementary Figure 1 Sequence alignment of methyltransferases MppJ and LmbW that methylate analogous substrates, phenylenolpyruvate and **2**/**3**, respectively. The alignment was generated using MAFFT web tool version 7.397. The identical residues are highlighted in rectangles. Positions of residues in active site of the proteins as depicted in Fig. 2c and d are highlighted with a red asterisk. Residues required for the common proposed mechanism, reflecting the common α-keto(enol)-carboxylic moiety of Ppy and **2**, are highlighted with a blue rectangle; residues in the putative active site of LmbW that differ from the corresponding homologous residues in published Ppy binding site, reflecting the difference between Ppy (aromatic ring) vs. **2** (heterocyclic carboxylic moiety) are highlighted with a green rectangle. GenBank No: MppJ - AAU34201.1 [https://www.ncbi.nlm.nih.gov/protein/AAU34201.1], LmbW - ABX00619.1 [https://www.ncbi.nlm.nih.gov/protein/ABX00619.1]
